# Diagnostic Yield of Genetic Disorders in Children with Hip Dysplasia Mimicking Bilateral Legg-Calvé-Perthes Disease

**DOI:** 10.3390/diagnostics16142293

**Published:** 2026-07-22

**Authors:** Beyhan Tüysüz, Nilay Güneş, Timur Yıldırım, Hasan Karakaş, Büşra Kasap, Hilal Onur, Dilek Uludağ Alkaya, Sezgin Şahin, Mehmet Müfit Orak, Gazi Zorer, Sebuh Kuruğoğlu, Özgür Kasapçopur

**Affiliations:** 1 Department of Pediatrics, Medical Faculty, Istanbul Atlas University, Istanbul 34408, Turkey; 2 Department of Pediatric Genetics, Cerrahpaşa Medical Faculty, Istanbul University-Cerrahpaşa, Istanbul 34098, Turkey; nilay.gunes@iuc.edu.tr (N.G.); busra.kasap93@gmail.com (B.K.); onurhilal52@hotmail.com (H.O.); dilek.uludagalkaya@iuc.edu.tr (D.U.A.); 3 Baltalimani Bone Diseases Training and Research Center, Department of Orthopedics and Traumatology, Medical Faculty, University of Health Sciences, Istanbul 34470, Turkey; drtimuryildirim@gmail.com; 4 Department of Pediatric Endocrinology, Medical Faculty, Kastamonu University, Kastamonu 37150, Turkey; drhsnkarakas@gmail.com; 5 Department of Pediatric Rheumatology, Cerrahpaşa Medical Faculty, Istanbul University-Cerrahpaşa, Istanbul 34098, Turkey; drsezginsahin@gmail.com (S.Ş.); ozgurkc@iuc.edu.tr (Ö.K.); 6 Department of Orthopedics and Traumatology, Medical Faculty, Istanbul Atlas University, Istanbul 34408, Turkey; doktor@mehmetmufitorak.com; 7 Independent Researcher, Istanbul 34158, Turkey; gzorer@gazizorer.com; 8 Department of Pediatric Radiology, Cerrahpaşa Medical Faculty, Istanbul University-Cerrahpaşa, Istanbul 34098, Turkey; sebuh.kurugoglu@iuc.edu.tr

**Keywords:** *COL2A1*, *COL9A1*, *COL9A3*, *COL11A1*, *COL11A2*, *RPL13*, *EIF2AK3*, *DNAJC21*, *ARSK*, Legg-Calvé-Perthes disease

## Abstract

**Background**: Pathogenic variants in genes that cause skeletal dysplasias may, instead of producing classic findings, present in children with a phenotype whose hip radiographs resemble bilateral Legg-Calvé-Perthes disease (LCPD). **Objectives**: This study aims to investigate the efficacy of genetic diagnosis in children with waddling gait or joint pain and radiological evidence of hip dysplasia mimicking bilateral LCPD. **Methods**: Forty children with bilateral femoral head dysplasia from 36 families were included in the study. Exome sequencing was performed, and all identified variants were confirmed within the families by Sanger sequencing. **Results**: Twelve pathogenic or likely pathogenic variants were identified: six in *COL2A1*, two in *COL9A1*, and one each in *RPL13*, *EIF2AK3*, *DNAJC21*, and *ARSK*; six are novel. The diagnostic yield was 33.3% (12/36) in 12 families. Additionally, variants of uncertain significance (VUS), proposed as causative, were detected in five families (5/36:13.9%): two in *COL11A1* and one each in *COL9A3*, *COL11A2*, and *ARSK*. Based on bilateral epiphyseal dysplasia of the femoral head, it was observed that seven families may be compatible with mild spondyloepiphyseal dysplasia and six families may have Stickler syndrome. Notably, among these, three children carrying closely localized pathogenic/likely pathogenic variants in *COL2A1* shared a novel phenotype characterized by short stature and bilateral irregular femoral heads. In four families, *EIF2AK3*, *DNAJC21*, and *ARSK* were also responsible for the ultra-rare disorders Wolcott-Rallison syndrome, bone marrow failure syndrome 3, and mucopolysaccharidosis 10, respectively. **Conclusions**: This study, for the first time, investigated the frequency of associated genes in a pediatric cohort with bilateral hip dysplasia resembling LCPD, providing important information for pathogenesis and differential diagnosis.

## 1. Introduction

Joint pain and fatigue during walking or running, or a waddling gait, are common reasons for presentation to pediatric outpatient clinics. If changes are detected on hip joint radiographs, the underlying causes may include rheumatic, infectious, or oncological conditions, as well as Legg-Calvé-Perthes disease (LCPD) and genetic disorders [1,2]. LCPD is avascular necrosis of the femoral head epiphysis that typically occurs between the ages of 4 and 8 years, is three to four times more common in boys than in girls, and is bilateral in 10–25% of cases. The radiologically diagnostic appearance is characterized by small, irregular, fragmented femoral head epiphyses [3,4,5,6]. Clinical and radiological findings of bilateral LCPD may overlap with disorders affecting epiphyseal growth, such as avascular necrosis of the head of the femur (ANFH), and skeletal dysplasias including, Beukes hip dysplasia, multiple epiphyseal dysplasia (MED), Stickler syndrome, mild forms of spondyloepiphyseal dysplasia (SED), and mucopolysaccharidosis (MPS) [7,8].

The pathogenesis of LCPD and ANFH is complex; some patients with heterozygous recurrent missense variants (p.Gly1170Ser or p.Gly630Ser) in *COL2A1* have been reported [3,4,5,6]. Although MIM (#150600) classifies LCPD as a disease caused by *COL2A1* mutations, the role of *COL2A1* in LCPD remains controversial [3]. Kannu et al. [9] identified two different mutations (p.Gly672Cys and p.Gly213Asp) in *COL2A1* in two children with bilateral LCPD. One of these children had additional findings such as short stature and midface hypoplasia, and the other had cleft palate; they, explicitly used the term “LCPD-like presentation” to distinguish them from idiopathic isolated LCPD. Studies examining the clinical phenotypes of patients with *COL2A1* mutations have shown that these patients may exhibit a wide range of clinical phenotypes such as early osteoarthritis of the hip, Stickler syndrome or mild type MED or SED congenita (SEDC) [10,11]. Type II collagen is the major protein of cartilage and, together with types IX and XI collagen, forms large fibrillar structures that are vital for the structure and function of cartilage [12]. The genetic pathogenesis of clinical phenotypes caused by *COL2A1*, as well as *COMP*, *COL9*, *COL11*, and *MATN3* which are involved in the extracellular matrix of cartilage was investigated in a comprehensive study. In this study, the role of variable expression, allelic heterogeneity, and other genetic and epigenetic factors was highlighted, suggesting that individuals carrying pathogenic variants in these genes may present with a phenotype predominantly affecting the proximal femoral heads, resembling LCPD, while showing only mild involvement of other joints and the spine, rather than the classic findings of the defined diseases; these phenotypes were termed MED and related disorders [13].

It is very difficult to clinically determine whether patients with bilateral hip dysplasia have underlying LCPD or other disorders. Isolated idiopathic LCPD and its mimicking phenotypes share features such as a waddling gait, joint pain, and similar radiological hip involvement. However, in other phenotypes there may also be additional skeletal or systemic findings that could be overlooked which require different follow-up and management approaches [3,4,14]. To the best of our knowledge, there are no studies on molecular yield rates in children with this group of disorders using exome sequencing approaches. This study aims to investigate the genetic diagnostic efficacy of exome sequencing in a well-defined cohort of children with hip and/or leg pain or waddling gait and radiological evidence of bilateral hip joint involvement mimicking LCPD.

## 2. Materials and Methods

### 2.1. Patients

The study included 40 children from 36 families who presented with joint pain or a waddling gait and bilateral femoral head epiphyseal involvement on radiographs, which could not be attributed to rheumatological or infectious causes. Patients with metaphyseal and vertebral involvement were excluded, while those with negligible metaphyseal or vertebral endplate irregularities were included. In addition, patients with typical clinical features and pathogenic variants in genes associated with MED (*COMP*, *COL9A1–A3* and *MATN3*, heterozygous; *SLC26A2* and *CANT1*, homozygous) were excluded from the study group. After exome sequencing analysis, reverse phenotyping was performed in all patients, regardless of the presence of a disease-causing variant, through joint evaluation at a single center by a pediatric orthopedist (TY), pediatric radiologists (SK), and pediatric geneticists (BT).

Physical examination findings, anthropometric measurements (weight, height, head circumference, arm span, sitting height and hand length), and skeletal deformities were recorded at initial and at annual follow-up examinations. The standard deviation score (SDS) for all anthropometric measurements was calculated using a national pediatric calculator based on national standards (https://www.ceddcozum.com). X-ray examinations of the skeleton, magnetic resonance imaging (MRI) of the hip, biochemical blood analysis, urinalysis, eye examination, hearing test, echocardiography and abdominal ultrasonography were performed. MRI findings suggestive of bilateral LCPD included proximal femoral epiphyseal abnormalities, subchondral linear low-signal intensity on T1-weighted images, and the presence of a double-line sign on T2-weighted images. Detailed histories and radiographs were also obtained from affected relatives.

### 2.2. Molecular Genetic Analysis

After comprehensive clinical characterization, exome sequencing was performed in 36 probands. Exome capture was carried out using the QIAseq Human Exome Kit (QIAGEN, Venlo, The Netherlands), followed by 101-base paired-end sequencing on the Illumina NovaSeq platform (Illumina, San Diego, CA, USA). Data processing included quality control using FastQC v0.12.1, alignment to the reference genome with BWA-MEM v0.7.17, variant calling with GATK v4.2.0.0, and annotation with ANNOVAR (version 2023 Mar 15). The mean exome read depth across the cohort was 129× (median: 118×; range: 74–233×). Between 94% and 98% of targeted regions achieved a minimum coverage depth of 20×. Variant calling focused on single nucleotide variants (SNVs) and small insertions and deletions (indels). Variant filtering was performed across the entire exome without restricting analysis to a predefined disease-specific gene panel. Variants demonstrating concordance with each patient’s clinical phenotype were prioritized for further interpretation. Rare variants were prioritized based on a minor allele frequency of less than 1% in public databases, a minimum quality score of 30, and a read depth of at least 10×. Variant classification was performed according to the American College of Medical Genetics and Genomics (ACMG) guidelines by integrating genetic, computational, and clinical evidence [15]. Population allele frequencies were assessed using in-house and Turkish genome databases (https://tgd.tuseb.gov.tr/en/; accessed on 16 March 2026), as well as gnomAD (v3) [16]. In silico prediction tools included Franklin Genoox (version 93), Varsome (version 13.17.1.0), SIFT v2.4, PolyPhen-2 (version 2.2.3), SpliceAI v1.3.1.0, CADD v1.7, and REVEL v1.3. For missense variants, a CADD Phred score of ≥15 and/or a REVEL score of ≥0.5 was considered supportive evidence for pathogenicity. Selected variants, including pathogenic, likely pathogenic variants and variants of uncertain significance (VUS), were confirmed by Sanger sequencing using an ABI PRISM 3500 genetic analyzer (Applied Biosystems, Foster City, CA, USA). After all variants were confirmed by Sanger sequencing in the proband, and in parental and family studies when available, variant inheritance was determined. Because five patients from five families had VUSs (variants of uncertain clinical significance) with very low population allele frequencies and clinical phenotypes consistent with those caused by related genes, these families were included in the uncertain diagnosis group based on overall clinical and genetic evidence.

## 3. Results

The study cohort comprised 40 children (31 males, nine females) from 36 unrelated families. The age of disease onset ranged from 1.5 to 12 years (median 4.5 years), and the initial findings in most patients were a waddling gait and hip and/or leg pain. Nine patients had mild to moderate short stature. Detailed clinical findings are presented in Supplementary Tables S1 and S2.

### 3.1. Molecular Findings

**Figure 1:**
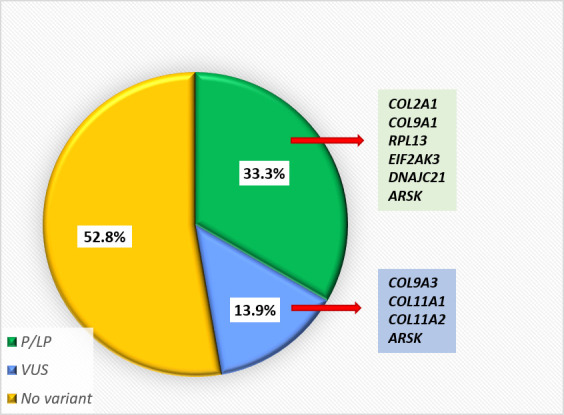
The pie chart shows the frequency of pathogenic/likely pathogenic (P/LP) variants and associated genes in 12/36 families. The diagnostic yield is 33.3%. The proportion of families with genes proposed as variants of uncertain significance (VUS) was 13.9% (5/36).

Twelve families had pathogenic or likely pathogenic variants; six of these variants were novel (Table 1). The diagnostic yield for disease-causing variants was 33.3% (12/36; 95% confidence interval (CI):18.6–51.0). Six of the 12 families had one child affected by a monoallelic *COL2A1* mutation (MIM#120140). Two different families had a biallelic mutation in *COL9A1*, one with two affected children. One family had a monoallelic mutation in *RPL13* (MIM#113703), while the other three families had biallelic mutations in *EIF2AK3* (MIM#604032), *ARSK* (MIM#610011; affecting two siblings), and *DNAJC21* (MIM#617048) (Figure 1).

In addition, VUSs were identified in five families (5/36; 13.9%). These VUSs were monoallelic state in *COL11A1* (MIM#120280) in two families, and biallelic in *COL9A3* (MIM#120270), *COL11A2* (MIM#120290) and *ARSK* in three families (Figure 1). In these families, the variants were considered proposed causal VUSs, resulting in an uncertain diagnosis because of their segregation within the family, their very low population frequencies, and clinical presentations consistent with these genes.

In 21 children from 19 families (52.8%), no pathogenic variant was found.

### 3.2. Clinical and Radiologic Features

#### 3.2.1. Patients with Pathogenic/Likely Pathogenic Variants and VUSs

Clinical and radiological findings were examined in 14 children from 12 families with pathogenic/likely pathogenic variants, along with five children with VUS (Table 1 and Table S1).

**Figure 2:**
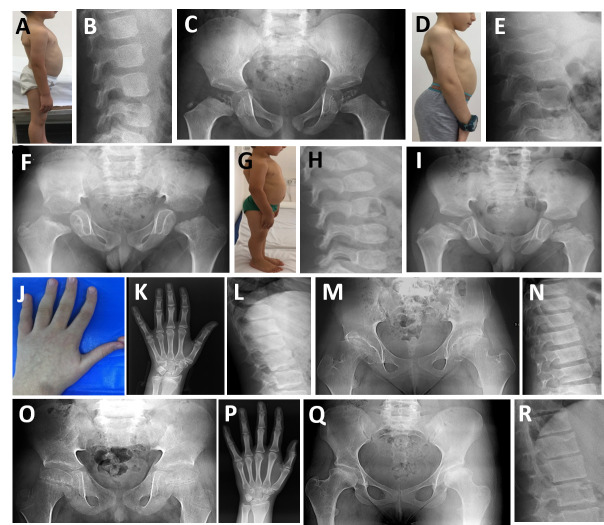
Patients with monoallelic disease-causing variants in *COL2A1*. P1 at age 4.5 (**A**–**C**), P2 at 7 (**D**–**F**), and P3 at 3.5 years of age (**G**–**I**). Note marked lumbar lordosis (**A**,**D**,**G**), mildly ovoid vertebrae (**B**,**H**), irregular vertebral endplates (**E**), and small, fragmented, irregular, or destroyed femoral heads with slightly broad and irregular proximal femoral metaphyses (**C**,**F**,**I**). P4, with SED and metatarsal shortening, at age 16: ulnar deviation of the second finger (**J**), normal metacarpal and phalangeal bones (**K**), slightly elongated vertebrae with irregular endplates (**L**), and irregularities of the femoral head and acetabulum with narrowing of the joint space and coxa valga (**M**). P5, with SED Stanescu type, at age 7, had mild platyspondyly (**N**), an irregular acetabulum, a slightly flattened femoral head, an irregular proximal femoral metaphysis, a broad femoral neck, and coxa valga (**O**) on radiographs. P6, with Stickler syndrome type I, at age 17, had long metacarpal bones (**P**), flat and irregular epiphyses of the femoral head (**Q**), and rectangular vertebral bodies (**R**).

Three patients (P1–P3) with monoallelic pathogenic/likely pathogenic variants (c.1492G>C, c.1510G>A, c.1538G>C) located in close proximity in *COL2A1* exhibited similar features, including short stature, severe lumbar lordosis, waddling gait, and joint pain (Table S1, Figure 2A,D,G). Their radiographs showed irregularities or destruction of the femoral heads, while the vertebral bodies were ovoid or slightly irregular (Figure 2B,C,E,F,H,I). P4, who had the recurrent c.823C>T variant in *COL2A1* associated with SED and metatarsal shortening, had normal height, joint pain, and ulnar deviation of the fingers (Figure 2J,K), as well as irregular vertebral endplates, femoral heads, and acetabulum (Figure 2L,M). P5, with a likely pathogenic missense variant in *COL2A1*, presented with finger stiffness, a waddling gait, leg pain, flattened proximal femoral epiphyses, and a broad femoral neck (Figure 2N,O). P6 and his affected father, with a known missense variant in *COL2A1*, had a waddling gait, short stature, severe myopia, arachnodactyly, and irregular proximal femoral epiphyses (Figure 2P–R).

**Figure 3:**
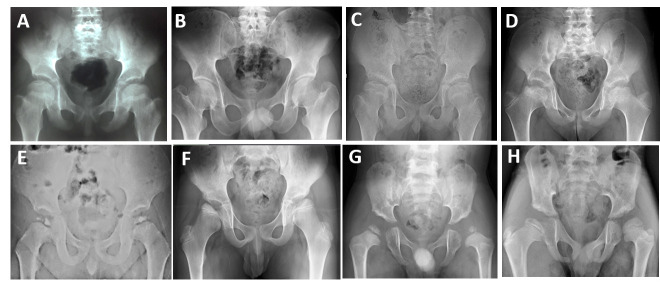
Radiographs of patients with biallelic variants in *COL9A1* (P7, P9) and *COL9A3* (P10) with Stickler types IV and VI are shown in the first line. P7 at 9 years (**A**) and P9 at 12 years (**C**) display bilateral, slightly irregular acetabula, flattened and slightly irregular proximal femoral epiphyses, and broad, shortened femoral necks with coxa valga. P7 at age 18 (**B**) shows a narrowed joint space, a slightly small femoral head and a shortened femoral neck with a high greater trochanter. In P10, at age 9 (**D**), an irregular acetabulum and a hypoplastic and irregular femoral head epiphysis, more pronounced on the right side and coxa vara, are noted. In the second line, pelvic radiographs of P11 and P12, who have a monoallelic variant in *COL11A1* are shown. Note the fragmentation of the proximal femoral epiphysis in 7-year-old P11 (**E**), and hypoplasia of the medial part of the proximal femoral epiphysis in 9-year-old P12 (**F**). P13, who has a biallelic variant in *COL11A2*, is shown in pelvic radiographs at ages 3 and 8. Note the oblique acetabulum and small, hypoplastic femoral head (**G**), as well as the coxa vara and straight femoral head (**H**).

Biallelic likely pathogenic frameshift and nonsense variants in *COL9A1* were detected in two siblings (P7, P8), and in P9, respectively. A biallelic VUS (in-frame deletion) in *COL9A3* was identified in P10 (Table 1). These patients had hip pain or a waddling gait, increased lumbar lordosis, and joint hypermobility, as well as mild scoliosis, myopia, and deafness compatible with Stickler syndrome types IV and VI (Table S1). Radiological features included an irregular acetabulum, flat and irregular femoral heads, and short femoral necks with coxa valga, observed in P7 and P9 at ages 9 and 12, respectively (Figure 3A,C). Radiographs taken at age 18 during follow-up of P7 revealed narrowed joint spaces, a slightly smaller femoral head, a shortened femoral neck, and a more superiorly positioned greater trochanter (Figure 3B). In P10, involvement of the femoral head epiphysis and acetabulum was more pronounced (Figure 3D).

Two monoallelic missense VUSs in *COL11A1* were identified in P11 and P12. Both children had a waddling gait and either bilateral fragmentation (Figure 3E) or hypoplasia of the medial part (Figure 3F) of the proximal femoral epiphysis, but no visual or hearing problems and midface hypoplasia. P13, with a biallelic missense VUS in *COL11A2*, had underdeveloped femoral epiphyses at the age of 3 (Figure 3G); by 8 years, flattened and irregular femoral heads had developed (Figure 3H).

**Figure 4:**
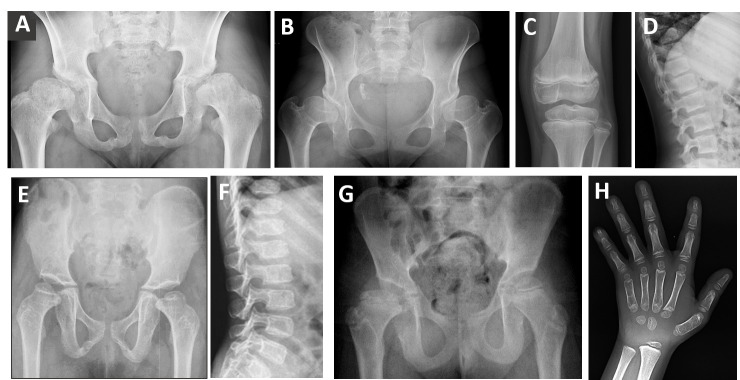
P14, with a monoallelic mutation in *RPL13*, was examined at ages 10 (**A**,**C**,**D**) and 17 (**B**) years. Note the slanted acetabulum and flat femoral head with a short femoral neck (**A**,**B**), while the metaphyses of the lower extremities and the vertebral bodies are normal (**C**,**D**). P15, with a biallelic *EIF2AK3* variant at age 7.5 years showed hypoplasia of the medial part of the capital femoral epiphysis, a horizontal acetabulum, coxa valga, and normal vertebral bodies (**E**,**F**). P16, with a biallelic *DNAJC21* mutation at age 10 showed an LCPD-like appearance of the right femoral head and a small, flattened femoral epiphysis on the left, with mild bilateral metaphyseal irregularity (**G**) and delayed bone age (**H**).

A heterozygous mutation of *RPL13* was detected in P14, who presented with a waddling gait and limb and hip pain at the age of 8 years; radiographs showed severe destruction of the epiphysis of the femoral head and a short, wide femoral neck (Figure 4A,B), while the vertebral bodies and metaphyses of the lower limb were normal (Figure 4C,D). A biallelic likely pathogenic variant in *EIF2AK3*, which causes early-onset diabetes mellitus (DM) and MED (MIM#226980), was detected in a girl (P15). This patient, who had bilateral proximal femoral epiphyseal hypoplasia, had neonatal DM in her history (Figure 4E,F). P16 had delayed bone age with an LCPD-like appearance of the right femoral head and a small, flattened femoral epiphysis on the left side (Figure 4G,H). In this patient, a biallelic mutation in *DNAJC21* leading to the very rare disease “bone marrow failure syndrome 3” (MIM#617052) was identified, and bone marrow failure developed during follow-up. Biallelic *ARSK* variants were found in three patients (P17-P19) from two families with bilateral epiphyseal irregularities in the femoral heads and metaphyseal striations. Three children with these gene variants leading to the very rare MPS type 10 (MIM#619698), did not have joint restriction, organomegaly, or radiologically specific dysostosis multiplex that would suggest MPS. Detailed clinical findings for these patients have been previously published by our group [22].

#### 3.2.2. Patients Without Variants Related to Hip Dysplasia Genes or Other Disorders

**Figure 5:**
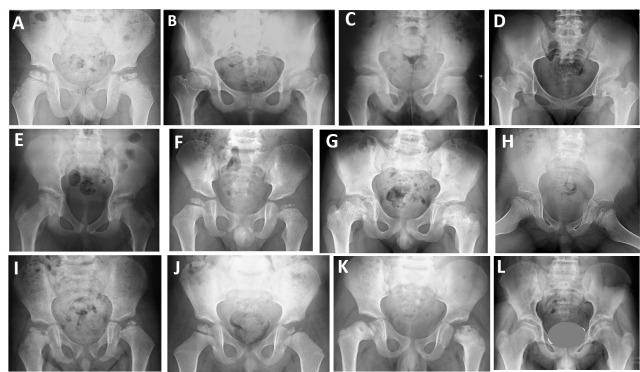
Radiographs of 12 patients without disease-causing variants. Patients with radiological features inconsistent with a specific skeletal dysplasia (**A**–**G**): severe irregularities of the proximal femoral epiphysis in P20 at 6 years of age (**A**); destruction of the femoral head in P21 at 16 years of age (**B**); flattening and irregularity of the bilateral femoral epiphyses in P22 at 9 years of age (**C**); a deformed femoral head and short femoral neck in P26 at 5 years of age (**D**); an irregular acetabulum and proximal femoral epiphyses in P33 at 11 years of age (**E**); a very small, irregular femoral head in P34 at 6 years of age (**F**); and irregular proximal femoral epiphyses in P38 at 8 years of age (**G**). Patients whose radiological features are consistent with LCPD (**H**–**L**): P23 at 3.5 years of age (**H**), P24 at 3 years of age (**I**), P32 at 4 years of age (**J**), and P36 at 4 years of age (**K**). Note the bilateral fragmented, small, irregular, or destroyed proximal femoral epiphyses. P28 at 15 years of age, with an ANFH phenotype, had irregular, flat proximal femoral epiphyses (**L**).

Of the 21 patients from 19 families without variants causing the disease, 18 were male, and the mean age at disease onset was 6 years (Table S2). Deep phenotyping of clinical findings, hip radiographs, and MRI scans was performed, revealing features associated with hip dysplasia ranging from mild to severe, inconsistent with a specific skeletal dysplasia phenotype (Figure 5A–G). The remaining nine patients showed a phenotype consistent with bilateral LCPD (Figure 5H–K), one with ANFH (Figure 5L), and three with features of Stickler syndrome.

## 4. Discussion

In this study, we performed exome sequencing on a cohort of pediatric patients with bilateral femoral head epiphyseal dysplasia resembling LCPD who did not have a specific clinical diagnosis. Disease-causing variants were detected in 33.3% of the families. In five families, VUS with a high likelihood of causing the disease were identified and proposed as causative, and these patients were also included in the discussion. Following molecular diagnosis, deep phenotyping was undertaken through multidisciplinary examination, to determine which genetic disorder the phenotype might be associated with.

The most common disease-causing variants were in *COL2A1*, found in six families. Heterozygous variants in *COL2A1* represent a large group of skeletal dysplasias, ranging from lethal forms to isolated osteoarthritis, and are listed as sixteen different phenotypes in MIM. Missense variants were most frequently observed (60%), including glycine substitutions within a Gly-X-Y repeat [19]. The three boys from unrelated families (P1–P3) presented here had missense variants (p.Gly498Arg, p.Gly504Ser, p.Gly513Ala) located close to each other in *COL2A1*, all involving glycine substitutions. The patients exhibited similar phenotypes, including mild- to moderate short stature (−2.7 to −4.2 SDS) with onset in early childhood, radiologically severe involvement of the femoral head epiphysis, and mild irregularities of the vertebral endplates. The p.Gly504Ser variant identified in P1 has previously been reported in a patient with SEDC [17]. The 2023 revision of the Genetic Skeletal Disorders nosology states that mild cases of SEDC within the group of type 2 collagenopathies may resemble MED, as well as mild chondrodysplastic osteoarthritis (Namaqualand-type hip dysplasia; MIM#604864) and Stanescu-type SED (MIM#616583) [8]. Although the recurrent p.Arg519Cys mutation reported in Namaqualand-type hip dysplasia (MIM#604864) is close to the variants found in our patients, their short stature and early childhood onset were not consistent with this dysplasia. They also differed from Stanescu-type SED by the absence of joint stiffness and the presence of short stature [23]. Our patients did not have vertebral or epiphyseal abnormalities as severe as those seen in SEDC, but all had short stature, which was inconsistent with the MED phenotype. We considered these patients to have a novel phenotype, a mild form of SEDC with similar clinical features and closely localized variants.

Stanescu-type SED (MIM#616583) is a very rare condition characterized by stiffness and pain in the hip and finger joints, a flattened acetabulum, a flattened and irregular femoral head, widening of the proximal femur, coxa valga, and metaphyseal irregularity [23]. We suspected that P5, in whom we found the novel variant (c.719G>T) in *COL2A1*, might have Stanescu-type SED because of stiffness in the finger joints, a waddling gait, leg pain, and similar pelvic radiological findings. However, a c.719G>A variant at the same protein position has also been reported in Stickler syndrome [24]. The recurrent Arg275Cys variant in *COL2A1* is known to cause SED with metatarsal shortening, formerly Czech-type SED (MIM#609162), which is characterized by symmetric spondyloarthropathy with symptoms that develop gradually during adolescence and primarily affect the hip joint [18]. This variant was identified in our patient (P4) who exhibited only severe hip involvement and subtle irregularities in the vertebral endplates on radiographs. There was ulnar deviation of the second finger, but no metacarpal shortening. Interestingly, the same variant has been reported in four patients from the same family, in whom only hip involvement and a short fourth fingers were described [25]. We also identified the pathogenic *COL2A1* variant, previously reported in two SEDC patients, in a girl (P6) and her father [19]. However, this patient and her father had severe hip involvement, myopia, and arachnodactyly compatible with Stickler syndrome type I (MIM#108300); the father underwent hip surgery at the age of 30 years. A recently published article stated that patients with genetically confirmed type 1 Stickler syndrome and a previous diagnosis of LCPD had retinal detachment, drawing clinicians’ attention to the possibility that patients with symptoms suggestive of LCPD may have underlying Stickler syndrome [14]. These observations highlight that the same or closely located variants in *COL2A1* can cause similar or variable clinical presentations, resulting in cases where hip dysplasia is prominent and other skeletal features are absent, instead of the classic SED findings.

Stickler syndrome is caused by pathogenic variants in monoallelic *COL2A1* (80%) and *COL11A1* (20%), and less frequently in biallelic *COL9A1*, *COL9A2*, and *COL9A3*. It is characterized by midface hypoplasia, myopia, hearing loss, and mild vertebral and epiphyseal involvement. In particular, the hip joint is affected, and LCPD-like changes leading to early-onset degenerative joint disease are typical [26].

A monoallelic disease-causing variant results in functional impairment of *COL9A1–A3*, causing MED types 2, 3, and 6, whereas biallelic loss-of-function variants in *COL9A1–A3* lead to a complete absence of collagen type IX and cause autosomal recessive Stickler syndrome types IV, V, and VI [13,26]. In this study, we detected novel biallelic nonsense or frameshift variants in *COL9A1*, resulting in complete absence of type IX collagen in three children from two unrelated families. One child also had a biallelic in-frame deletion VUS in *COL9A3.* These four patients had bilateral involvement of the proximal femoral epiphysis, as well as vision and hearing loss consistent with Stickler syndrome types IV (MIM#614134) and VI (MIM#620022) [26,27]. Radiographs of patients with *COL9A1* variants showed hip dysplasia including an irregular acetabulum, flattened and irregular proximal femoral epiphyses, and a short, wide femoral neck, as previously reported [27]. In the patient with the *COL9A3* VUS, the hip radiological findings were more pronounced than in both the patients with *COL9A1* presented here and those reported previously [28,29].

Monoallelic pathogenic *COL11A1* variants causing Stickler syndrome type II (MIM#604841) are mostly splicing and missense variants. We identified two different heterozygous missense variants, proposed as causative VUS in *COL11A1* in P11 and P12 from unrelated families. This syndrome presents with midfacial hypoplasia; 15–20% of patients have no visual or hearing problems, and mild SED findings are present in 25% of cases [26]. In our patients (P11 and P12), the hip joints were affected, but there was no flat midface or visual or hearing problems. A mild form of Stickler syndrome type II caused by *COL11A1* mosaicism was reported in the latest nosology of skeletal dysplasias [8]. Biallelic disease-causing variants in *COL11A2* cause autosomal recessive deafness (MIM#609706) or otospondylomegaepiphyseal dysplasia (MIM#215150), characterized by short stature, joint contractures and pain, premature osteoarthritis, platyspondyly, and large epiphyses [30]. A boy (P13) in our study with a proposed causative biallelic VUS in *COL11A2* had neither hearing loss nor platyspondyly with large epiphyses. He underwent surgery for developmental hip dysplasia in infancy; at age 6, his height was −1.9 SDS and he had only irregular proximal and distal femoral epiphyses. Recent studies on the genetics of hip dysplasia have suggested that the association between *COL11A1* and *COL11A2* and early-onset osteoarthritis is supported by evidence linking these genes to hip dysplasia [31,32].

Monoallelic pathogenic variants in *RPL13*, which encodes ribosomal protein L13 expressed in the growth plate, cause spondyloepimetaphyseal dysplasia, Isidor-Toutain type (MIM#618728), characterized by short stature, platyspondyly, and irregularities of the metaphyses and epiphyses of the lower extremities [33]. Interestingly, a patient with a mild phenotype showing only LCPD-like femoral epiphyseal changes and carrying the same pathogenic variant in *RPL13* as our patient (P14) has been reported [20].

Our results support the disease mechanisms proposed by Dennis et al. [13]. They emphasized that individuals carrying the same variants in genes involved in the extracellular matrix of bone, such as *COMP*, *COL2*, *COL9*, *COL11*, and *MATN3*, may exhibit clinical findings ranging from mild to severe due to reduced penetrance or other factors. We observed that epiphyseal dysplasias in long bones other than the proximal femur, and spinal involvement, which should be present in SED, are so mild that they can be overlooked or may be absent.

We also identified biallelic variants in *EIF2AK3*, *DNAJC21*, and *ARSK* that cause extremely rare syndromes in four families with hip dysplasia, instead of the typical syndromic features. Biallelic mutations in *EIF2AK3* cause “Early-onset DM and MED” (MIM#226980; Wolcott-Rallison syndrome), which is clinically variable and in some patients also affects the epiphyses [34]. In a girl (P15) with gait abnormalities and bilateral epiphyseal involvement of the proximal femur on hip radiograph, we detected a biallelic likely pathogenic variant in *EIF2AK3*; her history stated that she had DM. A biallelic *DNAJC21* variant causing “bone marrow failure syndrome 3” (MIM#617052) was detected in our patient, who had an LCPD-like appearance of the right femoral head and a small, flat femoral epiphysis on the left side, and who developed bone marrow failure during follow-up. One patient with the same variant as our patient, and with similar hip dysplasia, has been reported [21]. In three patients with bilateral epiphyseal and acetabular irregularity of the proximal femur, we identified biallelic *ARSK* variants (one classified as a VUS) causing type X MPS (MIM#619698), an extremely rare form of the disease. These patients, whose clinical findings we previously published [22], did not exhibit prominent signs of dysostosis multiplex; additionally, they had distinctive metaphyseal striations. Based on our experience with this group of extremely rare syndromes, patients presenting with hip involvement must be carefully screened for endocrine, hematological, and lysosomal diseases.

A limitation of this study is that twenty-one patients in our cohort could not be genetically diagnosed and genome sequencing was not performed in this group. Clinical findings in half of these patients were consistent with bilateral LCPD or ANFH. Another limitation of this study is the inability to conduct functional studies to evaluate gene or protein expression that would indicate the pathogenicity of VUSs or clinical phenotype variability.

In conclusion, variants causing the disease were identified in 33.3% of families with bi-lateral femoral head epiphyseal dysplasia without a specific clinical diagnosis. This study demonstrates that disorders resulting from mutations in *COL2A1*, *COL9A1*, *COL9A3*, *COL11A1*, *COL11A2*, *RPL13*, *EIF2AK3*, *DNAJC21*, and *ARSK* can initially present with hip dysplasia, without the classic findings of the diseases associated with these genes. Based on this observation, early genetic diagnosis may be crucial in preventing other life-threatening problems associated with these disorders. Furthermore, our results highlight the role of these genes in the differential diagnosis of patients with bilateral LCPD-like presentation and underscore their importance in the pathogenesis of morphological hip abnormalities.

## Figures and Tables

**Table 1:** Molecular findings and associated phenotypes in 17 families.

Family/ Patient Number	Gene (Transcript)	Variant (Protein Change)	ACMG Classification	Zygosity	Reference for Variants	MIM Phenotype/Number That May Be Associated with Clinical Features
**F1/P1**	*COL2A1* (NM_001844.5)	c.1510G>A (p.Gly504Ser)	Pathogenic (PS4, PM1, PP2, PM2, PM5, PP3)	Monoallelic	Xu et al. [17]	A mild form of SED congenita?
**F2/P2**	*COL2A1* (NM_001844.5)	c.1538G>C (p.Gly513Ala)	Likely pathogenic (PM1, PP2, PM2, PM5, PP3)	Monoallelic	This study	
**F3/P3**	*COL2A1* (NM_001844.5)	c.1492G>C (p.Gly498Arg)	Likely pathogenic (PM1, PP2, PM2, PM5, PP3)	Monoallelic	This study	
**F4/P4**	*COL2A1* (NM_001844.5)	c.823C>T (p.Arg275Cys)	Pathogenic (PS4, PP1, PM2, PP3, PP2)	Monoallelic	Markova et al. [18]	SED, metatarsal shortening/609162
**F5/P5**	*COL2A1* (NM_001844.5)	c.719G>T (p.Gly240Val)	Likely pathogenic (PM1, PP2, PM2, PM5, PP3)	Monoallelic	This study	SED, Stanescu type/ 616583
**F6/P6**	*COL2A1* (NM_001844.5)	c.2059G>A (p.Gly687Ser)	Pathogenic (PS4, PP1, PM1, PM2, PM5, PP2, PP3)	Monoallelic	Barat Houari et al. [19]	Stickler syndrome I/ 108300
**F7/P7, P8**	*COL9A1* (NM_001851.6)	c.1491delT (p.Pro498Leufs Ter133)	Likely pathogenic (PVS1, PM2)	Biallelic	This study	Stickler syndrome IV/614134
**F8/P9**	*COL9A1* (NM_001851.6)	c.151C>T (p.Gln51Ter )	Likely pathogenic (PVS1, PM2)	Biallelic	This study	Stickler syndrome IV/614134
**F9/P10**	*COL9A3* (NM_001853.4)	c.1081_1083del (p.Gly361del)	VUS (PM2,PM4)	Biallelic	This study	Stickler syndrome VI/ 620022
**F10/P11**	*COL11A1* (NM_080629.3))	c.2644C>T (p.Arg882Trp)	VUS (PM2, PP3)	Monoallelic	This study	Stickler syndrome II/604841
**F11/P12**	*COL11A1* (NM_001854.4)	c.2768C>T (p.Pro923Leu)	VUS (PM2,PP3)	Monoallelic	This study	Stickler syndrome II/604841
**F12/P13**	*COL11A2* (NM_080680.3)	c.4312G>T (p.Gly1438Cys)	VUS (PM2, PP3)	Biallelic	This study	Mild form SED or developmental hip dysplasia?
**F13/P14**	*RPL13* (NM_000977.4)	c.477+1G>A -	Pathogenic (PVS1, PM2, PS4)	Monoallelic	Reinsch et al. [20]	SED, Isidor-Toutain type/618728
**F14/P15**	*EIF2AK3* (NM_004836.7)	c.1126G>T (p.Glu376Ter)	Likely pathogenic (PVS1, PM2)	Biallelic	This study	Early-onset DM and MED (Wolcott-Rallison syndrome)/226980
**F15/P16**	*DNAJC21* (NM_001012339.3)	c.983+1G>A -	Pathogenic (PVS1, PM2, PM3)	Biallelic	D’Amours et al. [21]	Bone marrow failure syndrome 3/617052
**F16/P17**	*ARSK* (NM_198150.3)	c.1254G>T (p.Met418Ile)	VUS (PM2,PP3)	Biallelic	Alkaya et al. [22]	MPS10/619698
**F17/P18, P19**	*ARSK* (NM_198150.3)	c.427G>T (p.Glu143Ter)	Likely pathogenic (PVS1, PM2)	Biallelic	Alkaya et al. [22]	MPS10/619698

**Abbreviations:** F family, P patient, DM Diabetes mellitus, MED multiple epiphyseal dysplasia, MIM Mendelian inherited man, MPS mucopolysaccharidosis, SED spondyloepiphyseal dysplasia, VUS variants of uncertain significance.

## Data Availability

The data presented in this study are available on request from the corresponding author due to privacy and ethical restrictions related to patient data.

## Supplementary Materials

The following supporting information can be downloaded at: https://www.mdpi.com/article/10.3390/diagnostics16142293/s1, Table S1: Clinical and radiological features of the patients with found pathogenic and likely pathogenic variants, and variants of uncertain significance; Table S2: Clinical and radiological features of the patients without disease-causing variants or VUS.

## Abbreviations

The following abbreviations are used in this manuscript:

ANFH	Avascular necrosis of the head of the femur
CI	Confidence interval
DM	Diabetes mellitus
LCPD	Legg-Calvé-Perthes disease
MED	Multiple epiphyseal dysplasia
MPS	Mucopolysaccharidosis
MRI	Magnetic resonance imaging
OSMED	Otospondylomegaepiphyseal dysplasia
SDS	Standard deviation score
SED	Spondyloepiphyseal dysplasia
SEDC	Spondyloepiphyseal dysplasia congenita
VUS	Variants of uncertain significance
