# Supplementary material for: Diagnostic Yield of Genetic Disorders in Children with Hip Dysplasia Mimicking Bilateral Legg-Calvé-Perthes Disease
Source: Diagnostics (Basel). 2026 Jul 22;16(14):2293. doi: 10.3390/diagnostics16142293 (PMC13409581; doi:10.3390/diagnostics16142293)
Supplement: Supplementary file 1 [file diagnostics-16-02293-s001.zip › diagnostics-4279073-supplementary.pdf]

Table S1: Clinical and radiological features of the patients with found pathogenic and likely pathogenic variants, and variants of uncertain significance

**Gene /Zygosity**	***COL2A1*/ Monoallelic**						***COL9A1* / Biallelic**			***COL9A3* / Biallelic**
**MIM phenotype/number associated with clinical features**	**Spondyloepiphyseal dysplasia resembling MED**			**SED with metatarsal shortening/ 609162**	**SED, Stanescu type/ 616583**	**Stickler type I/108300**	**Stickler type IV/614134**			**Stickler type VI/ 620022**
**Family number**	1	2	3	4	5	6	7		8	9
**Patient number**	1	2	3	4	5	6	7	8	9	10
**Gender (F/M)**	M	M	M	F	M	F	F	M	M	M
**Family history**	-	-	-	-	-	+ (father)	+	+	-	-
**Age at initial/last visit (years)**	4.5/8.5	7/9	3/7.5	10.6/16	7/7	17/18	9/18	5.2/19	12/15	4.2/9.5
**Initial sign**	Short stature, waddling gait	Short stature, waddling gait	Short stature, genu varum	Waddling gait, genu valgum	Waddling gait, joint pain	Waddling gait	Lumbar lordosis, joint pain	Scoliosis	Waddling gait	Waddling gait
**Age of initial sign (years)**	3	2	1.5	5	5	2	6	5	4	3
**Height SDS (first /last visit)**	-3.2 /-2.7	-4.1/ -4.2	-3.8 /-3.4	1.2/ 1.07	+1/ +1	-1.6/ -1.7	-0.6/ -2.4	0.2/ -1.3	-0.5 /0.2	-1.2 /0.9
**Clinical diagnosis**										
Waddling gait	+	+	+	+	+	+	-	-	+	-
Difficulty climbing stairs	+	+	+	+	-	-	-	-	-	-
Joint pain (hip and/or leg)	+	-	+	+	+	+	+	+	-	+
Fatigue to long distance walking	+	+	+	+	+	-	-	-	-	-
Genu varum/valgum	-/-	-/-	+/-	-/+	-/-	-/-	-/-	-/-	-/-	-/+
Brachydactyly	-	-	-	-	-	-	-	+	+	+
Arachnodactyly	-	-		-	-	+	-	-	-	-
Limited elbow joint	+	+	-	+	-	-	+	+	-	+

Finger stiffness<br>Joint hypermobility<br><br>Lumbar lordosis<br>Pes planus<br>Premature osteoarthritis/	-<br>-<br><br>+<br>-<br>-	-<br>-<br><br>+<br>-<br>-	-<br>-<br><br>+<br>+<br>-	-<br>-<br><br>-<br>-<br>+	+<br>-<br><br>-<br>-<br>-	-<br>-<br><br>-<br>-<br>+	-<br>Finger, knee<br>+<br>-<br>-	-<br>Knee<br><br>+<br>+<br>-	-<br>Finger, knee<br>-<br>-<br>-	-<br>-<br><br>-<br>+<br>-
Surgery	-	-	-	Hip	-	-	-	-	-	-
Myopia<br>Other eye features	-<br>-	-<br>-	-<br>Vision loss	-<br>-	-<br>-	+<br>-	+<br>-	+<br>-	-<br>Retinal detachment	+<br>-
Hearing loss	-	-	-	-	-	-	+ (SN)	+ (SN)	-	+ (SN)
Other features	-	-	-	-	-	-	-	-	-	-
**Radiological features**										
Delayed/irregular carpal ossification	+/-	+/+	+/+	+/+	-/-	-/+	-/+	-/+	+/+	+/+
Metacarpal/phalangeal bones	N/N	N/N	N/N	Long/N	Long/N	Long/long	-/-	-/-	-/-	Short/short
Acetabular roof<br>Irregular/destructive/small femoral head<br>Irregular proximal femoris metaphysis<br>Short/broad femoral neck<br>Coxa vara/valga	N<br>+/-/+<br>-<br>-/+<br>-/-	N<br>-/+/-<br>+<br>+/+<br>-/-	N<br>+/-/+<br>+<br>-/+<br>-/+	N<br>+/-/-<br>+<br>-<br>-/+	Irregular<br>+/-/-<br>-<br>-/+<br>-/+	Irregular<br>+/-/-<br>-<br>-<br>-/+	N<br>+/-/-<br>-<br>+/+<br>-/+	N<br>+/-/-<br>-<br>+/+<br>-/+	N<br>+/-/-<br>-<br>+/+<br>-/+	N<br>+/+/-<br>+<br>-/-<br>-/-
Metaphyseal irregularity<br>Mild platyspondyly or irregular vertebral endplates /Scoliosis	-<br>-/-	-<br>+/-	-<br>+/-	-<br>+/-	-<br>+/-	-<br>-/-	-<br>-/+	-<br>+/+	-<br>-/-	-<br>+/-

Table S1: Continuous

**Gene/Zygosity**	***COL11A1*/ Monoallelic**		***COL11A2* / Biallelic**	***RPL13* / Monoallelic**	***EIF2AK3*/ Biallelic**	***DNAJC21*/ Biallelic**	***ARSK*/ Biallelic**		
**MIM phenotype/number associated with clinical features**	Stickler type II/604841		MED-like phenotype	SEMD,Isidor-Toutain type/ 618728	MED-with early onset DM/ 226980	Bone marrow failure syndrome 3/ 617052	Mucopolysaccharidosis type 10/619698		
**Family number**	10	11	12	13	14	15	16	17	
**Patient number**	11	12	13	14	15	16	17	18	19
**Gender(F/M)**	M	M	M	F	F	M	M	F	M
**Family history**	-	+ (father)	-	-	-	-	-	+	+
**Age at initial/last visit (years)**	2.8/7	7.8/13	3.1/8	10/18	7.5	3/10.5	11.7/12.7	11.8/18.1	9.1/14.1
**Initial sign**	Waddling gait	Waddling gait	Bilateral DDH	Waddling gait	Waddling gait	Genu valgum	Waddling gait	Hip pain	Knee pain
**Age of initial sign (years)**	2.5	5	3	8	4	1	9	9	9
**Height SDS (first/last visit)**	0.1/ 0.4	-0.2/ -0.6	-1.9/ -1.9	-0.3/ -1.2	-6.2	-1.5/ -2.3	-0.4/ 0.1	-1.1/ -2.3	-0.1/ -0.7
**Clinical features**									
Waddling gait	+	+		+	+	+	+	+	+
Difficulty climbing stairs	-	-	-	-	-	-	-	+	-
Joint pain (hip and/or leg)	-	+	-	+	-	-	+	+	+
Fatigue to long distance walking	-	+	-	-	-	-	+	-	-
Genu varum/valgum	-/-	-/-	-/-	+/-	-/-	-/+	-/-	-/-	-/-
Brachydactyly	-	-	+	+	-	+	-	+	-
Arachnodactyly	-	-	-	-	-	-	-	-	-
Limited elbow joint	+	-	-	-	+	+	+	-	+
Finger stiffnes	-	-	-	-	-	-	-	-	-

Joint hypermobility<br>Lumbar lordosis<br>Premature osteoarthritis	-<br>-<br>-	Elbow<br>+<br>-	-<br>-<br>-	Hand<br>-<br>-	-<br>-<br>-	+<br>+<br>-	+<br>-<br>-	+<br>+<br>-	-<br>-<br>-
Surgery	-	-	DDH	-	-	-	-	-	-
Myopia<br>Other eye features	-	-	-	-	+	-	-	-	-
Hearing loss	-	-	-	-	-	-	-	-	-
Other features	-	Pectus carinatu m	-	Pes planus	Neonatal diabetes mellitus type-1	Amelogenesis imperfecta, bone marrow failure, cupped ears	-	-	-
**Radiological features**									
Delayed /irregular carpal ossification	+/-	-/-	+/-	+/+	-/-	+/-	+/+	+/+	+/-
Metacarpal/ phalangeal bones	N/N	N/N	Short/N	N/N	N/N	N/N	N/N	N/N	N/N
Acetabular roof<br>Irregular/dysplastic/ small femoral head<br>Short/broad femoral neck<br>Coxa vara/valga	N<br>+/+/+<br>-/-<br>-/+	N<br>+/+/+<br>-/-<br>-/-	Slanted<br>+/-/+<br>+/-<br>+/-	Slanted<br>+/+/-<br>+/+<br>+/-	Horizonta l<br>+/+/-<br>-/-<br>-/+	N<br>+/+/-<br>-/+<br>-/+	Slanted/ irregular<br>+/-/-<br>+/-<br>-/-	N<br>+/-/-<br>+/-<br>-/-	Slanted/ irregular<br>+/-/-<br>+/-<br>-/-
Metaphyses	N	N	N	N	N	N	Vertical striations	Vertical striation s	Vertical striations
Mild platyspondyly or irregular vertebral endplates	-	+	-	-	-	-	+	+	+

ANFH: avascular necrosis of the femoral head; DDH: developmental dysplasia of hip; LCPD: Legg-Calve-Perthes disease; MED: Multiple epiphyseal dysplasia; N:Normal; SED: spondyloepiphyseal dysplasia; SEMD: Spondyloepimetaphyseal dysplasia; SN: Sensorineural

Table S2: Clinical and radiological features of the patients without disease-causing variants or VUS

**Family number**	**18**	**19**	**20**	**21**	**22**	**23**	**24**	**25**	**26**	**27**
**Patient number**	**20**	**21**	**22**	**23**	**24**	**25**	**26**	**27**	**28**	**29**
**Gender (F/M)**	M	F	M	M	M	M	F	M	M	F
**Family history**	-	+	-	-	-	-	-	-	-	-
**Age at initial /last visit (years)**	6/10.2	16/16	9/9	3.5/3.5	3/7.7	6.4/6.4	4.3/10.5	1/15	13.9/18	3.9/8.4
**Initial symptoms**	Waddling gait, hip pain	Hip pain	Waddling gait	Waddlin g gait	Hip pain	Waddling gait	Genu valgum	Short stature	Genu valgum, knee pain	Hip and leg pain
**Age of initial sign (years)**	5	5	7	3.5	3	5	2	1	12	3.5
**Clinical diagnosis**	MED-like	MED-like	MED-like	Bilateral LCPD	Bilateral LCPD	MED-like	MED-like	Stickler syndrome	ANFH	Bilateral LCPD
**Height SDS (initial/last visit)**	-0.4/0.5	-3.2	-1.1	2.2	-1/-0.6	-0.1	-1.5/-2.6	-1.6/-2.8	0.9/0.6	-1.7/-1.1
**Clinical features**										
Waddling gait	+	+	+	+	-	+	+	-	-	-
Difficulty climbing stairs	-	**+**	+	-	-	**+**	**+**	**-**	**-**	**-**
Joint pain (hip and/or leg)	+	+	-	+	+	+	-	+	+	+
Fatigue to long distance walking	-	**+**	-	-	-	**+**	**+**	**-**	**-**	**-**
Genu varum/valgum	-/-	-/-	-/+	-/+	-/-	-/-	-/+	-/+	-/+	-/-
Brachydactyly	-	-	-	-	-	-	-	+	-	-
Limited elbow joint	-	+	-	-	-	-	+	+	-	-
Joint hypermobility	-	-	-	-	+	-	-	+	-	-
Lumbar lordosis	+	-	-	+	-	-	+	-	-	-
Premature osteoarthritis	-	-	-	-	-	-	+	-	+	-
Surgery	-	+ (father)	-	-	-	DDH	-	-	Knee	-
Eye examination	-	Bilateral cataract	-	-	-	-	-	Myopia	+	-

Hearing loss	-	-	+	-	-	-	-	+	-	-
Other features	-	-	-	-	-	-	-	-	-	-
**Radiological features**										
Delayed/irregular carpal ossification	+/+	-/+	-/+	-/-	+/-	-/-	+/+	+/+	+/+	+/-
Metacarpal/phalangeal bones	-/-	-/-	+/-	-/-	-/-	-/-	-/-	+/-	-/-	-/-
Acetabular roof<br>Irregular/dysplastic/flat femoral head<br>Short/broad femoral neck<br>Coxa vara/valga	N<br>+/-/+<br>-/-<br>-/-	N<br>+/+/+<br>+/+<br>-/-	Horizontal<br>+/-/+<br>-/-<br>-/-	N<br>+/+/-<br>-/-<br>-/-	N<br>+/+/-<br>-/-<br>-/+	N<br>+/+/+<br>+/+<br>-/-	Irregular<br>+/-/+<br>+/+<br>+/-	N<br>-/-/+<br>+/+<br>-/-	N<br>+/-/+<br>-/-<br>-/-	N<br>+/+/-<br>-/-<br>-/-
Irregular knee epiphyses<br>Metaphyseal irregularity/ vertical striations	+<br>-/-	-<br>-/-	-<br>-/-	-<br>-/-	+<br>-/-	-<br>-	+<br>-	+<br>-	+<br>-	+<br>-
Mild metaphyseal irregularity	**+**	**-**	**-**	-	**-**	-	**-**	**+**	**-**	**-**
Mild platyspondyly or irregular vertebral endplates /Scoliosis	-/-	+/+	-/-	-/-	-	-	+	+	-	+

Table S2: Continuous

**Family number**	**28**	**29**	**30**	**31**	**32**	**33**		**34**	**35**	**36**	
**Patient number**	**30**	**31**	**32**	**33**	**34**	**35**	**36**	**37**	**38**	**39**	**40**
**Gender (F/M)**	M	M	M	M	M	M	M	M	M	M	M
**Family history**	-	-	-	-	-	Monozygotic twins		-	-	+	+
**Age at initial/ last visit (years)**	10.8/15.4	7.3/7.6	2.9/4.1	11/11	6.1/6.1	3.9/4.5	3.9/4.5	8.7/10.5	8.5/8.5	6.5/8.5	4.5/4.5
**Initial symptoms**	Waddling gait	Waddling gait	Genu valgum	Waddling gait, leg pain	Knee and hip pain	Joint pain	Joint pain	Waddling gait	Waddling gait	Waddling gait	Waddling gait
**Age of initial sign (years)**	8	6	2	10	5.5	2	3	3	7	5	4
**Clinical diagnosis**	Bilateral LCPD	Bilateral LCPD	Bilateral LCPD	MED-like	MED-like	Bilateral LCPD	Bilateral LCPD	MED-like	Bilateral LCPD	Stickler syndrome	
**Height SDS (first/last visit)**	-0.3/-0.6	2/0.4	0.8/0.4	-0.7	-2.4	-0.8/-0.9	-0.1/-0.4	-1.7/-1.7	+1.5	+1	0.8
**Clinical features**											
Waddling gait	+	+	-	+	-	+	-	+	+	+	+
Difficulty climbing stairs	-	-	-	-	-	-	-	-	-	+	+
Joint pain (hip and/or leg)	+	+	-	+	+	+	+	-	-	-	
Fatigue to long distance walking	+	+	-	-	-	-	-	-	-	-	-
Genu varum/valgum	-	-	-/+	-	-	-	-	-	-	-	-
Brachydactyly	-	-	-	-	-	-	-	+	-	-	-
Limited elbow joint	-	-	-	-	-	-	-	+	-	+	-
Joint hypermobility	-	Hand	-	Hand	Hand	-	-	-	-	+	+
Lumbar lordosis	-	-	-	+	+	-	-	-	+	+	+

Premature osteoarthritis	-	-	-	-	+	-	-	-	-	-	-
Surgery	-	-	-	-	-	-	-	-	-	-	-
Hearing loss	-	+	-	-	-	-	-	+	-	+	+
Other features	-	--	-	-	Hypothyroidism	-	-	-		Cleft palate	Cleft palate
**Radiological features**											
Delayed/irregular carpal bones	-/+	-/-	+/-	-+	+/-	-/-	-/-	-/+	-/-	-/-	-/-
Brachydactyly	-	-	-	-	-	-	-	+	-	-	-
Acetabular roof	Irregular	N	N	Irregular	Irregular	N	N	N	Irregular, horizontal	N	N
Irregulari/dysplastic/flat femoral head	-/+/-	+/+/-	+/-/+	+/-/-	+/+/+	+/+/-	+/+/-	+/-/+	+/+/+	+/+/-	+/+/-
Short/broad femoral neck	+/+	-/-	+/+	-	+/+	+/+	+/+	+/+	-/-	-/-	-/-
Coxa vara/valga	+/-	-/-	-/-	-/-	-/-	-/-	-/-	+/-	-/-	-/-	-/-
Irregular knee epiphyses	-	-	-	-	+	-	-	-	-/-	-	-
Metaphyseal vertical striations	-	-	-	-	-	-	-	-	-	-	-
Metaphyseal irregularity	-	-	-	-	-	-	-	-	-	-	-
Mild platyspondyly or irregular vertebral endplates /scoliosis	+/-	-/-	-/-	+/-	+/-	-/-	-/-	+/-	+/-	-/-	-/-

ANFH: avascular necrosis of the femoral head; DDH: Developmental dysplasia of hip; LCPD: Legg-Calve-Perthes disease; MED: Multiple epiphyseal dysplasia; N:Normal.
